# Effect of maturation time on dormancy and germination of *Citrullus colocynthis* (Cucurbitaceae) seeds from the Arabian hyper-arid deserts

**DOI:** 10.1186/s12870-017-1209-x

**Published:** 2017-12-22

**Authors:** Ali El-Keblawy, Hatem A. Shabana, Teresa Navarro, Sameh Soliman

**Affiliations:** 1 Department of Applied Biology, Faculty of Science, University of Sharjah, P. O. Box, 27272 Sharjah, United Arab Emirates; 2 Department of Biology, Faculty of Science, Al-Arish University, Al-Arish, Egypt; 3 Sharjah Seed Bank and Herbarium, Sharjah Research Academy, P.O. Box 60999, Sharjah, United Arab Emirates; 4 Departmento de Biología Vegetal, Universidad de Málaga, P. O. Box 59, 29080 Málaga, Spain; 5 Department of Medicinal Chemistry, College of Pharmacy, University of Sharjah, P. O. Box, 27272 Sharjah, United Arab Emirates

**Keywords:** Cucurbitaceae, Day-length, Dormancy, Germination requirements, Maternal effect, Time of fruit maturation

## Abstract

**Background:**

Light and temperatures of germination greatly affect germination of several Cucurbitaceae species. Environmental conditions prevailing at seed maturation time can affect dormancy and germination requirements. *Citrullus colocynthis* seeds have a deep dormancy. This perennial prostrate shrub grows all over the year in the arid Arabian deserts. We explored if seed dormancy and germination requirements of *C. colocynthis* depend on time of fruit collection. Matured seeds were collected at five different times during 2014/2015 year from a population around Dubai city. Fresh seeds were germinated at three temperature regimes in both continuous darkness and alternating 12 h light/12 h darkness. Impacts of seed storage and other treatments on germination were applied on seeds collected in March and exhibited deep dormancy.

**Results:**

March collected seeds almost did not germinate in both light and dark at the three temperatures, but those of the other collections responded differently to both light and temperatures. At the lowest temperatures, seeds of all collections did not germinate in light, but those of June, October and December collections germinated in dark. There were negative correlations between final germination and seed length, width, mass and coat thickness. Physical scarification, water soaking and seed storage did not break dormancy of March collection.

**Conclusions:**

Germination of *C. colocynthis* is very sensitive to light and incubation temperature as well as to the environmental conditions associated with the time of seed maturation. It is important to investigate the effects of environmental factors prevailing during seed maturation under controlled conditions to understand exact reasons for unusual seed dormancy and germination requirements of *C. colocynthis*, which seems to be very sensitive to maternal environment.

## Background

Seed dormancy is a temporary failure of a viable seed to complete germination under normally favorable physical environmental conditions [1]. It enables seeds to delay germination until the environment is favorable to subsequent seedling survival. Seed germination in the unpredictable desert environments is usually controlled by adverse climatic conditions, such as drought and/or high temperatures [2]. Many desert plants produce seeds with different types and levels of dormancy that could only be broken once they are exposed to proper environmental signals. In many cases, such signals could be coincided with the proper time of seedling establishment [3, 4].

It has been reported that environmentally induced parental effects can influence the phenotypic expression of morphological, physiological, and several life-history traits in plants [5–11]. For example, seeds matured at different seasons have different temperature and light requirements during germination [7, 12–14]. Several studies have reported that autumn or winter-matured seeds germinated significantly greater at higher temperatures and in continuous light, compared to spring- or summer-matured seeds [12, 14–16]. In addition, Cone & Spruit [17] found that seeds of *Arabidopsis thaliana* harvested in winter were more sensitive to light than those harvested in summer. The seasonal timing of seed maturation and dispersal can in turn determine the season of seed germination and germination rate and consequently the overall life history [14].

The photoperiod during seed maturation is particularly a reliable indicator of a growth season. Photoperiod and light quality prevailing during seed maturation can affect seed dormancy and subsequent germination in several species. In general, germinability is promoted with short day regimes in many species such as *Chenopodium polyspermum* [18], *C. album* [19], *Portulaca oleracea* [13, 20], *Beta vulgaris* [21], *Amaranthus retroflexus* [22] and *Aegilops kotschyi* [23]. However, short days resulted in greater dormancy in fewer other species, such as *Avena fatua* and *A. sterilis, Polygonum monspeliensis* and *Carrichtera annua* [7].

Temperature is another very important factor experienced during seed maturation that affects seed dormancy [7]. Generally, seeds produced at higher temperatures have lower dormancy (i.e., higher germinability) in many species [13, 16, 23–28]. It has been proposed that light, temperatures and other environmental factors prevailing during seed development can affect seed dormancy by affecting seed chemical composition and provisioning (e.g. mineral, photosynthetic and phytohormone resources) and through affecting structure and thickness of the seed coat [8, 28, 29]. In addition, environmental cues like light and temperature can change the tissue-specific localization of GA biosynthesis [30], which is well known to play a crucial role in regulating seed germination [31, 32].

Several studies have documented the importance of light and temperatures during germination process in triggering germination of several Cucurbitaceae species. For example, darkness is a requirement for seed germination of many species, such as *Citrullus lanatus*, *Cucurbita maxima*, *Lagenaria siceraria*, *Benincasa hispida* and *Momordica harantia* [33], *Citrullus lanatus* var. Sugar Baby [34], and *C. lanatus* var. *citroides* [35]. In addition, seed germination of other species of Cucurbitaceae is sensitive to the germination temperature. For example, melon germination sharply declined from almost 100% to zero when the temperatures were below the optimum [36]. In addition, germination of the Sugar Baby watermelon in darkness was nearly 100% at 20–40 °C, but decreased sharply at 15 °C and 42.5 °C [34]. Further, there was no germination in *Citrullus lanatus var. citroides* at day/night temperatures between 10/5 and 15/10 °C regardless of light regimes [37].

*Citrullus colocynthis* (bitter apple) is a desert plant of Cucurbitaceae. It is a small perennial herb with prostrate or climbing annual stem and perennial rootstocks. The plant could propagate by both seeds and vegetative buds on the rootstocks. This species has many medicinal benefits against different ailments including diabetes type II [38] and breast cancer [39]. It possesses anti-inflammatory and anti-bacterial compounds that may help to fight the related diseases [40]. In addition, *C. colocynthis* produces big amount of oily seeds that could be converted to low cost biodiesel [41], which is similar to that of Jatropha seeds [42]. In the UAE, several researchers reported the flowering time of *C. colocynthis* in the period from November to July [43, 44]. However, the extensive surveys in the UAE showed that the plants of this species are evergreen and can flower and produce fruits all over the year in few sandy places.

Mature seeds of *C. colocynthis* collected from Negev desert [45], UAE desert [46] and Iranian desert [47, 48] did not germinate without treatments. Dormancy of this species was attributed to the mechanical barrier of testa (i.e., physical dormancy), but not to the presence of allelochemicals in the seed testa that might inhibit the germination. None of these studies mentioned the time of seed maturation and collection. As *C. colocynthis* is fruiting all over the year in some habitats of the UAE, the present study aimed to assess the impacts of time of fruit collection on dormancy level, and factors that might trigger germination, such as light and temperature. We hypothesized that environmental factors prevailing during seed development and maturation, such as day length and temperatures, could affect dormancy level, and light and temperature requirements during seed germination. As seeds of one collection (March) did not germinate, another aim of the study was to assess the impact of different treatments, such as dry storage, soaking in water and physical scarification on dormancy breakage and germination requirements of this seed lot.

## Methods

### Study area

The Northern Emirates of the United Arab Emirates (UAE) is generally hot and dry with a sub-tropical arid climate, which is warm in winter and hot humid in summer. The region is characterized by two distinctive seasons: a long humid season (April to November) with very high temperatures and a short season (December to March) with mild to warm temperatures and light rainfall. The mean daily temperature ranges between 12.1 °C in January and about 42 °C in June–August. Temperatures can reach up to 48 °C in summer. The average annual rainfall in the coastal area is 120 mm [49].

The climatic data of the study area during the study year shows that the coolest temperatures were in January (minimum and maximum are 16.4 °C and 24.8 °C, respectively), while the hottest were in July and August (minimum and maximum are 32.2 °C and 41 °C, respectively). The average relative humidity ranged from 30.7% in May to 58.2% in January. The study year was very dry; total amount of rainfall received during the whole growing season (October 2014 – June 2015) was only 26.9 mm (72% of them in January). December has the shortest day length (10.4 h), while the longest was in June (13.5 h) (Table 1).

**Table 1:** Monthly variation in temperatures, relative humidity, precipitation and day length in nearest (Nadd ash Shiba) meteorological station, Dubai, to the studied population

Month	Temp. (°C)			Humidity (%)			rainfall (mm)		Photoperiod (day length in hours)*
	Max.	Min.	Avg.	Max.	Min.	Avg.	Avg.	Sum	
July-2014	41.13	32.16	36.58	72.52	23.84	50.00	0.00	0.0	13.3
August	40.97	32.23	36.71	71.68	24.32	49.77	0.00	0.0	12.8
September	39.47	30.07	34.70	78.00	22.70	55.03	0.00	0.0	12.0
October	36.52	27.58	31.84	69.81	22.39	48.58	0.02	0.5	11.3
November	29.67	22.07	25.90	66.73	27.83	49.47	0.00	0.0	10.6
December	26.52	17.94	22.06	74.68	29.81	55.32	0.02	0.5	10.4
January-2015	24.81	16.39	20.58	80.13	26.97	58.52	0.61	19.5	10.47
February	27.96	19.43	23.79	73.32	22.86	50.36	0.01	0.3	11.2
March	29.19	20.55	24.84	74.10	22.61	51.10	0.06	1.8	12.0
April	33.47	23.57	28.40	71.80	21.97	48.37	0.14	4.3	12.4
May	39.00	28.39	33.55	50.65	11.32	30.68	0.00	0.0	13.2
June	40.00	30.83	35.40	64.40	21.83	44.23	0.00	0.0	13.4

*Data are extracted from “Time and date website 2015”, Dubai [62]

### Seed collection

Fully ripened yellow fruits of large uniform sizes were collected five times throughout the 2014/2015 growing season (mid of October and December 2014, early March, and mid of April and June 2015) from a wild population of *C. colocynthis* growing around Dubai city, north of the UAE. Generally, yellowish colour of fruits was used as an indicator for fruit ripening. To diminish the effect of genetic variation, we used 20–30 individuals permanently tagged to collect fruits of the different collections. Immediately after collection, seeds were extracted manually from the fruits and then washed with water, dried, and stored in brown paper bags at room temperatures. Fresh seeds were germinated within 10–15 days after their collection.

For each collection, the average seed mass was determined by weighing three replicates, each of 50 seeds. In addition, average seed length, width and height and seed coat thickness was assessed in 50 seeds of each collection.

### Germination experiment

In order to assess light and temperature requirements during germination of *C. colocynthis*, seeds were germinated (incubated) in three programmed incubators adjusted to a daily night/day temperature regime of 15/25 °C, 20/25 °C and 25/35 °C in both continuous darkness and alternating 12 h darkness/ 12 h light (hereafter referred as dark and light, respectively). A dark condition was achieved by wrapping the Petri dishes with two layers of aluminum foil. Seeds were germinated in 9-cm tight-fitting Petri dishes containing one disk of Whatman No. 1 filter paper with 10 ml of distilled water. Four replicate dishes, each with 25 seeds, were used for each treatment. A seed was considered to be germinated when the radicle had emerged. Germinated seedlings were counted and removed every alternate day for 30 days following seed soaking. Seeds incubated in the dark were checked only once after 30 days. Therefore, they were not exposed to any light during the incubation period.

To assess possible reasons for high dormancy (no germination) recorded for March collection, several dormancy breakage treatments were assessed. These include dry storage, soaking in water and physical scarification. Physical scarification was performed on one-month stored seeds by cutting a part of the coat at the seed side with a nail clipper, without harming the underlying endosperm. Both scarified and non-scarified seeds were soaked for 48 h and incubated in light and temperature regimes as mentioned above.

To assess the effect of long term on germination, seeds of March and December were stored in brown paper bags at room temperatures for one year and then incubated in light and temperature regimes as mentioned above. The storage conditions mimic the natural conditions of after-ripening of buried seeds.

### Data analyses

The rate of germination was estimated using a modified Timson index of germination velocity = ΣG/t, where G is the percentage of seed germination at 2-day intervals and t is the total germination period [16]. The maximum possible value for our data using this germination rate index (GRI) was 50. The higher the value, the more rapid the germination. The germination rate was only calculated for seeds incubated under light conditions.

Three-way ANOVA was used to assess the significance of the main factors (collection time, and temperature and light of incubation) and their interactions on final germination. The same test was used to assess the effect of seed storage, light condition and incubation temperature and their interactions on final germination of March seeds that were after-ripened (stored) for one year. Two-way ANOVA was used to assess the impact of maternal habitat and incubation temperature and their interaction on the germination rate index (GRI). The same test was used to assess the effect of seed storage and incubation temperature and their interaction on germination rate index of March collection. Pearson correlation coefficient (r) was used to assess the significance of the relationship between germination in light and in dark and different seed traits (e.g., seed length, width, height and mass and seed coat thickness. Tukey test (Honestly significant differences, HSD) was used to estimate least significant range between means. The germination rate was log-transformed and germination percentages were arcsine-transformed to meet the assumptions of ANOVA. This transformation improved normality of the distribution of the data. All statistical methods were performed using SYSTAT, version 13.0.

## Results

### Germination of fresh seeds

There were significant effects (*P* < 0.001) of time of seed collection, incubation temperature and light condition and their interactions on final germination of *C. colocynthis* seeds (Table 2). Seeds collected in March didn’t germinate at all. The overall germination of April collected seeds (52%) was significantly greater than that of both June and October collected seeds (27.8% and 36.7%, respectively), but was significantly lower than that of December collection (74%). In addition, germination had significantly increased with the increase in incubation temperature and was significantly greater in dark than in light.

**Table 2:** Results of three-way ANOVA showing the effects of time of seed collection, and temperature and light of incubation on final germination of *Citrullus colocynthis* seeds from fresh fruits

Source	df	Mean Squares	F-Ratio	*p*-Value
(A) Final germination percentage (arcsine transformed)				
Collection time (CT)	4	4.597	548.952	<0.001
Temperature (T)	2	2.503	298.902	<0.001
Light (L)	1	0.816	97.477	<0.001
CT x T	8	0.689	82.238	<0.001
CT x L	4	0.356	42.532	<0.001
T x L	2	1.502	179.344	<0.001
CT x T x L	8	0.524	62.534	<0.001
Error	90	0.008		
(B) Germination rate index (log transformed)				
Collection time (CT)	4	23.709	22,256.983	<0.001
Temperature (T)	2	63.248	59,373.865	<0.001
CM x T	8	5.947	5582.507	<0.001
Error	45	0.001		

**Fig. 1:**
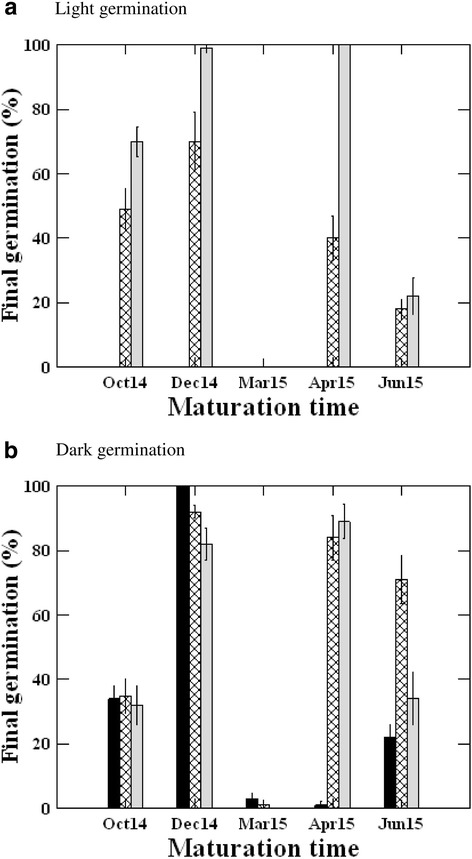
Effect of time of seed maturation, and incubation temperature on final germination (mean ± SE) of fresh *Citrullus* in *colocynthis* seeds in (**a**) light and (**b**) dark photoperiods

The effect of the interaction between time of fruit collection, light condition and incubation temperature on seed germination was significant (*P* < 0.001, Table 2). Few seeds of the March collection germinated in both light and dark at the three tested temperatures. However, germination of seeds from the other collections responded differently to both incubation temperature and light condition. At15/25 °C, seeds of all the collections did not germinate in light and seeds of April collection did not germinate in dark. At the same temperature, seeds of June, October and December collections germinated to 22%, 34% and 100% in dark, respectively (Fig. 1).

In light, germination at 25/35 °C was significantly greater than at 20/30 °C in October, December and April seeds, but not in June seeds. In dark, however, there was no significant difference in final germination of seeds of all the collections at 20/30 °C with that at 25/35 °C, except for June collected seeds. Interestingly, for December collection, germination reached almost 100% at the highest temperature in light, but at lowest temperature in dark. These results indicate that germination in light requires higher temperatures, but germination in darkness seems to be independent on temperature regime; it depends more on the time of seed collection (Fig. 1).

**Fig. 2:**
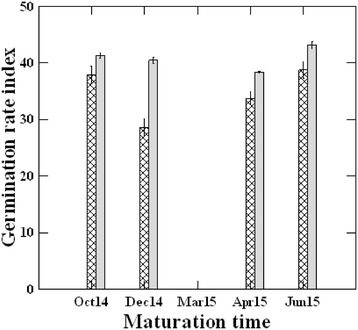
Effect of time of seed maturation, and temperature of incubation on germination rate index (mean ± SE) of *Citrullus colocynthis* fresh seeds. Hatched and light bars are for 20/30 °C and 25/35 °C, respectively. No germination happened at 15/25 °C

The effects of seed collection time and incubation temperature and their interaction on germination rate index were significant (*P* < 0.001, Table 2. Germination of all seed collections was significantly faster at 25/35 °C than at 20/30 °C. At 15/25 °C, no germination occurred in light and germination rate index was not calculated for germination in dark (Fig. 2).

Seeds of March and June are bigger, heavier and with thicker seed coat, but attained significantly lower germination, compared to seeds of the other collections. For seeds of all collections, there were negative correlations between final germination in light and in dark and different seed traits, including seed length, width, mass and coat thickness. However, this relationship was significant in case of light germination (*P* < 0.05), but not for dark germination, *P* > 0.05, Table 3).

**Table 3:** Final germination percentage of fresh and one-year stored seeds in light (LG) and dark (DG) and some seed traits of the different collections of *Citrullus colocynthis*

Seed Collection	Storage	LG	DG.	Length (mm)	Width (mm)	Height (mm)	Mass of 50 Seeds (g)	Coat Thickness (mm)
Oct 2014	Fresh	39.7	33.7	0.53	0.34	1.50	0.90	0.17
Dec 2014	Fresh	56.3	91.3	0.57	0.31	1.55	0.93	0.20
March 2015	Fresh	0.0	1.3	0.80	0.42	2.05	2.21	0.31
April 2015	Fresh	46.7	58.0	0.55	0.32	1.62	0.98	0.22
June 2015	Fresh	13.3	42.3	0.78	0.48	2.02	2.38	0.30
Dec 2014	Stored	4.5	1.0	0.80	0.42	2.05	2.21	0.31
March 2015	Stored	42.3	53.3	0.57	0.31	1.55	0.93	0.20
r for DG of fresh seeds				−0.6	−0.58	−0.63	−0.61	−0.55
r for DG of fresh seeds				−0.92*	−0.88*	−0.93*	−0.93*	−0.88*

r = correlation coefficients of the relationships between germination in light and in dark with different seed traits. *: r is significant at *P* = 0.05

### Effects of dry storage

#### March seeds

Storage for one year did not affect the dormancy level of seeds matured in March; no germination occurred in fresh seeds and those stored for one year (data are not shown).

#### December seeds

**Fig. 3:**
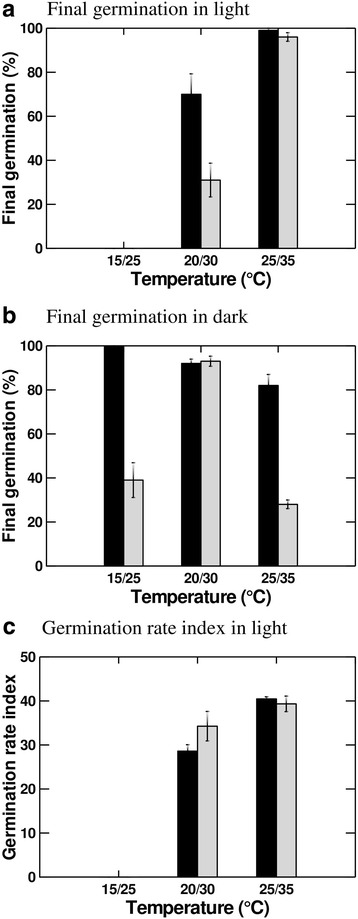
Effect of two years storage, and temperature on (**a**) final germination in light (mean ± SE), (**b**) final germination in dark and (**c**) germination rate index of *Citrullus colocynthis* seeds matured in December. Dark and light bars are for fresh and stored seeds, respectively

Three-way ANOVA showed significant effects for the main factors (seed storage, incubation temperature and light condition) and their interactions on final germination of *C. colocynthis* seeds matured in December (*P* < 0.001, Table 4). Storage resulted in the reduction of the germination, but the reduction depended on incubation temperature and light condition. Germination in dark of the stored seeds was significantly lower at lower (15/25 °C) and higher (25/35 °C) temperatures, compared to that of fresh seeds. At intermediate temperature (20/30 °C), there was no significant difference in dark germination between fresh and stored seeds. In addition, germination in light did not differ significantly between fresh and stored seeds at 25/35 °C, but was significantly greater in fresh, compared to stored seeds, at 20/30 °C (Fig. 3).

**Table 4:** Results of three-way ANOVA showing the effects of storage, and temperature and light of incubation on final germination of *Citrullus colocynthis* seeds matured in December

Source	Df	Mean Squares	F-Ratio	*p*-Value
(A) Final germination percentage (arcsine transformed)				
Storage (S)	1	2.039	140.604	0.000
Temperature (T)	2	1.185	81.703	0.000
Light (L)	1	0.935	64.482	0.000
S * T	2	0.132	9.089	0.001
S * L	1	0.466	32.132	0.000
T * L	2	3.521	242.828	0.000
S * T * L	2	0.710	48.930	0.000
Error	36	0.015		
(B) Germination rate index (log transformed)				
Storage (S)	1	0.013	1.825	0.193
Temperature (T)	2	34.467	4931.527	0.000
S x T	2	0.023	3.231	0.063
Error	18	0.007

### Effects of scarification and water soaking on march seeds

Neither physical scarification (scars in seed coat) nor water soaking resulted in any improvement in germination of March seeds. Almost no germination occurred at the different temperatures in both light and dark after seed scarification and water soaking (data are not represented).

## Discussion

The present study showed that *C. colocynthis* seeds germinated very well in both light and dark at moderate and higher temperatures (20/30 and 25/35 °C). At lower temperature (15/25 °C), germination of all collections was completely inhibited in light, but reached to ~ 100% in dark for December collection. This result is consistent with a trend observed in many species of the family Cucurbitaceae; seeds require warm temperatures for successful germination, but fail to germinate at low temperatures [36, 50]. For example, whereas melon seeds germinated to very high level at high temperatures, their germination fallen to zero when the temperatures were below 15 °C [36]. Similarly, germination of the Sugar Baby watermelon in dark was nearly 100% at optimal temperatures (20–40 °C), but decreased sharply at lower temperatures (15 °C) [34]. The failure of melon seeds to germinate at low temperatures has been attributed to low levels of endogenous gibberellin, impermeability of the testa to gases, and deficiency of the growth potential of the embryo [51]. In addition, it has been proposed that the seed coat-imposed dormancy at low temperature in melon seeds is the combined effect of lower amount of oxygen diffusion through the seed coat and greater embryo sensitivity to oxygen deficiency, rather than to physical constraints of radicle break-through or impairment of imbibition [52]. In *C. colocynthis*, the ability of June, October and December seeds, but not those of March and April, to germinate in dark at the low temperature regime indicate that temperature requirement has a phylogenetic basis as well as maternally induced effect [34, 36, 50].

Seeds of many species of the family Cucurbitaceae are negatively photoblastic; i.e., their germination is inhibited in light [34]. For example, Nakamura et al. [33] reported that germination of *C. lanatus*, *Cucurbita maxima*, *Lagenaria siceraria*, *Benincasa hispida* and *Momordica harantia* was hindered by continuous white light at 20–30 °C. However, germination of *Cucumis sativus*, *C. melo* was inhibited in light only at 20 °C. Our results indicated that *C. colocynthis* germination was completely inhibited in light at 15/25 °C, but ranged between as low as 1% for April collection to 100% for December collection in dark. At higher temperatures, however, seeds germinated equally well in light and dark. This result further supports a significant role for maternal environment in regulating germination in light.

Time of seed development and maturation affects seed dormancy and germinability of several species [5, 6, 8]. Several studies have reported that even little variation in temperatures during seed maturation can influence seed germination and dormancy. For example, caryopses of *Aegilops ovata* maturated at higher temperatures (28/22 °C) germinated significantly greater than those matured on plants grown at relatively lower temperatures (15/10 °C) [53]. Similarly, seeds of *Amaranthus retroflexus* matured on plants grown at 27/22 °C attained greater germination than those matured at 22/17 °C [54]. In *C. colocynthis*, seeds collected in March didn’t germinate at all in both light and dark at all the tested temperatures. Seeds of December and April attained the highest germination among the different seed collections. Despite the great variation in the germination of the three collections, they were all developed at different times during winter. It was noticed that fruits of both December and April collections developed and matured within less than a month, but those of March required more than two months for full maturation (Ali El-Keblawy, unpublished data). The average temperatures during fruit development and maturation was cooler for fruits collected at early March (average minimum and maximum temperatures of January and February was 17.9 °C and 26.4 °C, respectively), compared to average temperatures of fruits matured at mid of December (average minimum and maximum temperatures of November and December was 22.1 and 29.7 °C, respectively) and those matured at mid of April average minimum and maximum temperatures of March and April was 22.1 and 31.3 °C, respectively) (Table 1). This indicates that the lower temperatures might be responsible of the great dormancy observed in March seeds.

In Cucurbitaceae, it is known that physiological dormancy is attributed to the presence of inhibitors or variable concentrations of growth factors within the seed coat, while physical dormancy is associated with the presence of physical factors regulated by the intact seed coat [35, 55]. In *C. colocynthis*, seeds from deserts of southeast of Iran showed less than 5% germination, but physical and chemical scarifications significantly increased the germination [48]. Similarly, Menon et al. [46] found that physical scarification followed by soaking for 48 h of *C. colocynthis* seeds from a UAE population resulted in a higher germination, compared to non-treated seeds. However, Koller et al. [45] reported that matured seeds from Southern Negev desert did not germinate under a wide range of experimental conditions. Those authors didn’t attribute such lower germination to the mechanical barrier of seed coat or the presence of allelochemicals, but to the presence of an inner seed membrane. Similarly, our study indicated that neither physical scarification nor water soaking and their combination was able to stimulate the germination of dormant seeds of *C. colocynthis* that were collected in March. However, non-treated seeds of other collections reached very high level of germination without any treatments. This result further supports the hypothesis that maternal effect plays a significant role in controlling dormancy and germinability of *C. colocynthis*.

Day length during seed development can also affect seed coat structure, thickness and composition [41, 56]. For example, long days promote thicker, harder coats that reduced seed germinability of several species [19, 57, 58]. Conversely, seeds matured during the short days have water permeable seed coats and germinated to higher level [6]. In *C. colocynthis,* however, seeds matured during shortest days (December) and those matured at longer days (April, June and October; have day lengths more than 12 h, Table 1) attained higher germination, at least at 20/30 and 25/35 °C, whereas seeds of March that matured at day lengths intermediate between the other collections didn’t germinate at all at the different temperatures in both light and dark. This indicates that seed coat attributes mediated through day length cannot explain the germination variation in *C. colocynthis*.

Storage of *C. colocynthis* fresh seed from an Indian population resulted in reduction of the germination from 72.9% for fresh seeds to only 16.4% after one year [59]. This was attributed to the increase of seed coat hardening that limited the gas exchange between the embryo and surrounding atmosphere [59]. In the present study, one-year storage of dormant seeds collected in March didn’t result in any alleviation in the dormancy level. However, storage of December seeds resulted in a significant reduction in the dark germination at both low and high temperatures, but not at the moderate temperatures. In addition, stored seeds germinated also to almost 100% in light at the high temperatures. Such result indicates that storage didn’t affect seed viability, but might changed phytochrome sensitivity in dark [60, 61].

### Conclusions

The overall results indicated that germination behavior in *C. colocynthis* is very sensitive to light and temperature during seed soaking as well as to the environmental conditions associated with the time of seed maturation. However, the exact mechanisms controlling such sensitivity are not clear. Since day length, temperature and other climatic factors vary simultaneously through the year, it is very important to investigate the effect of these factors individually and in combinations under controlled experimental conditions on seed dormancy of *C. colocynthis*. Such experiments could help to understand the differential sensitivity of the seeds to light condition and incubation temperatures. In addition, assessing different assimilates, such as phytohormones and enzymes as well as anatomical investigation for different parts of seed coat and under laid membrane might help to understanding mechanisms underlying maternal effects on germination behavior in *C. colocynthis*.

## Abbreviation

GRI: Germination rate index
