# Correction to: Effect of maturation time on dormancy and germination of Citrullus colocynthis (Cucurbitaceae) seeds from the Arabian hyper-arid deserts

**DOI:** 10.1186/s12870-018-1262-0

**Published:** 2018-03-20

**Authors:** Ali El-Keblawy, Hatem A. Shabana, Teresa Navarro, Sameh Soliman

**Affiliations:** 1 Department of Applied Biology, Faculty of Science, University of Sharjah, P. O. Box, 27272 Sharjah, United Arab Emirates; 2 Department of Biology, Faculty of Science, Al-Arish University, Al-Arish, Egypt; 3 Sharjah Seed Bank and Herbarium, Sharjah Research Academy, P.O. Box 60999, Sharjah, United Arab Emirates; 4 Departmento de Biología Vegetal, Universidad de Málaga, P. O. Box 59, 29080 Málaga, Spain; 5 Department of Medicinal Chemistry, College of Pharmacy, University of Sharjah, P. O. Box, 27272 Sharjah, United Arab Emirates

## Correction

Following publication of the original article [1] author Hatem Shabana wrote to say that one of her affiliations was missing. The affiliation is as follows:

Departmento de Biología Vegetal, Universidad de Málaga, P. O. Box 59, 29080 Málaga, Spain
